# Modeling and observation of mid-infrared nonlocality in effective epsilon-near-zero ultranarrow coaxial apertures

**DOI:** 10.1038/s41467-019-12038-3

**Published:** 2019-10-02

**Authors:** Daehan Yoo, Ferran Vidal-Codina, Cristian Ciracì, Ngoc-Cuong Nguyen, David R. Smith, Jaime Peraire, Sang-Hyun Oh

**Affiliations:** 1 Department of Electrical and Computer Engineering, University of Minnesota, Minneapolis, MN 55455 USA; 2 Department of Aeronautics and Astronautics, Massachusetts Institute of Technology, Cambridge, MA 02139 USA; 3 Center for Biomolecular Nanotechnologies, Istituto Italiano di Tecnologia, Via Barsanti 14, 73010 Arnesano (LE), Italy; 4 Center for Metamaterial and Integrated Plasmonics, Department of Electrical and Computer Engineering, Pratt School of Engineering, Duke University, Durham, NC 27708 USA

**Keywords:** Nanophotonics and plasmonics, Metamaterials, Nanocavities, Sub-wavelength optics

## Abstract

With advances in nanofabrication techniques, extreme-scale nanophotonic devices with critical gap dimensions of just 1–2 nm have been realized. Plasmons in such ultranarrow gaps can exhibit nonlocal response, which was previously shown to limit the field enhancement and cause optical properties to deviate from the local description. Using atomic layer lithography, we create mid-infrared-resonant coaxial apertures with gap sizes as small as 1 nm and observe strong evidence of nonlocality, including spectral shifts and boosted transmittance of the cutoff epsilon-near-zero mode. Experiments are supported by full-wave 3-D nonlocal simulations performed with the hybridizable discontinuous Galerkin method. This numerical method captures atomic-scale variations of the electromagnetic fields while efficiently handling extreme-scale size mismatch. Combining atomic-layer-based fabrication techniques with fast and accurate numerical simulations provides practical routes to design and fabricate highly-efficient large-area mid-infrared sensors, antennas, and metasurfaces.

## Abstract (web summary)

Nonlocality is typically studied in simplified 2D or symmetric 3D structures and the ability to perform nonlocal simulations for complex 3D structures is lacking. Here the authors examine non-local optical effects in small gaps and its effect on the optical response of a coaxial metamaterial in the mid-IR.

## Introduction

The ability of metal nanostructures to localize light below the diffraction limit and enhance the optical field via surface plasmons—collective oscillations of free charge carriers—forms the basis of nano-optics and plasmonics^1–3^. Ultraconfined light in nanoscale apertures, tips, and gaps has been harnessed for surface-enhanced spectroscopy^4^, super-resolution imaging^5^, optical trapping^6^, and nonlinear optics^7^. With continual advances in top–down nanofabrication and bottom-up synthesis techniques, researchers can manufacture large-scale metal structures with critical dimensions below even 1 nm^8^. One of the most efficient geometries to realize plasmonic field confinement and enhancement is a nanometric gap formed between two metallic elements^9–11^. Pushing these gaps down to sub-nanometer distances in a precisely controlled manner enabled researchers to investigate nonlocal electrodynamics^11–16^ and light-induced quantum tunneling effects^17–19^ in the visible and near-infrared regime. Novel applications of resonant nanogap structures and antennas are expanding toward the mid-infrared (MIR) regime (typically 2–20 µm in wavelength), which is the emerging frontier showing great promise for biochemical sensing and spectroscopy^20–25^. However, nonlocal electrodynamics in MIR nanophotonic structures and its impact on the device design, ultimate performance (localization and field enhancement), and characterization accuracy have not yet been investigated, due to the significant challenges in both fabrication and numerical modeling. This work presents practical approaches to solve these challenges.

In general, nonlocal effects arise from the inhomogeneous nature of matter at the microscopic level. In metals, a strong component arises from intrinsic quantum properties of the electron gas, and can have a measurable impact even on large systems^12^. The most general linear relation between the electric field and the electric displacement vector in a homogenous medium can be expressed as:

1 $${\mathbf{D}}\left( {{\mathbf{r}},t} \right) = \varepsilon _0{\int} {dt\prime d{\mathbf{r}}\prime \varepsilon ({\mathbf{r}} - {\mathbf{r}}\prime ,t - t\prime ){\mathbf{E}}({\mathbf{r}}\prime ,t\prime )} ,$$

where *ε*(**r**, *t*) is the nonlocal dielectric tensor. This constitutive relation can be written as ${\mathbf{D}}\left( {k,\omega } \right) = \varepsilon _0\varepsilon (k,\omega ){\mathbf{E}}(k,\omega )$ in the Fourier domain. In the local response approximation (LRA), the wavelength is assumed to be much larger than the characteristic dimensions (the lattice spacing of a metal or the Thomas–Fermi screening length), hence the dielectric tensor is invariant with respect to the wavevector, that is $\varepsilon \left( {{\mathbf{r}},t} \right) = \varepsilon (t)\delta ({\mathbf{r}})$ in real space, with *δ* being the Dirac delta function. In this case, Eq. (1) can be then simplified as ${\mathbf{D}}\left( \omega \right) = \varepsilon _0\varepsilon (\omega ){\mathbf{E}}(\omega )$, which is no longer dispersive in space. In metal nanogap structures, however, light acquires an effective wavelength that can be comparable to characteristic microscopic dimensions and the spatial dispersion can become significant enough to cause experimental observations to deviate from the local model. Extremely localized surface plasmons in the nanogap between a gold nanoparticle and a mirror demonstrated the limitation of plasmonic field enhancement and the nonlocal effect in the visible and near-infrared regimes^12^. While a full quantum mechanical description of optical response is not yet possible for structures other than small clusters^26,27^, a semi-empirical hydrodynamic model has been successfully applied to describe electron–electron interactions in the limit of the Thomas–Fermi approximation in film-coupled nanoparticle systems at optical frequencies^12,28^.

To extend this method toward the longer-wavelength regime, in particular the mid-IR regime, it is necessary to address the increased size mismatch between the minimum feature dimensions and the free-space wavelength, that is over ten times larger than in the visible regime, presenting both simulation and fabrication challenges. It should also be noted that most existing works studied nonlocality in simplified two-dimensional (2-D) geometries or in specific (spherically or axially) symmetric three-dimensional (3-D) structures due to the computational burden. Practical applications, however, may involve arbitrarily shaped structures, often arranged in periodic arrays. Hence, the ability to perform nonlocal simulations for full 3-D structures with complex geometries is paramount. We overcome the simulation challenge of resolving rapid field variations over the Thomas–Fermi screening length (~0.1 nm; about 10^5^ times smaller than MIR wavelengths) with a fast and accurate 3-D computational method, the hybridizable discontinuous Galerkin (HDG) method, that accounts for nonlocal effects via a hydrodynamic model, while the fabrication challenge is overcome via atomic layer engineering. We quantify the nonlocal effect on both spectral resonance and transmission intensity in the mid-IR by comparing measurements from coaxial apertures with gap sizes of 1–10 nm with numerical calculations based on the hydrodynamic model. While previous work has concluded that nonlocality is responsible for limiting the field enhancement in a film-coupled nanoparticle system^12^, our aperture geometry, which harnesses extraordinary optical transmission (EOT)^29^, allows us to show that nonlocality can positively boost the transmission efficiency by effectively enlarging the gap width.

## Results

### ENZ mode and nonlocal response

It is not trivial to push the resonance wavelength of metal nanoparticle-based systems toward the mid-IR regime. Moreover, the gap-plasmon resonance of the film-coupled nanoparticle system is characterized via extinction measurements in reflection mode, but many practical applications in nanophotonics require optical transmission through sub-wavelength apertures. As a practically relevant model system to investigate the mid-IR nonlocality in transmission mode, we use coaxial nanoapertures^30–33^ that exhibit strong mid-IR resonances and can be made with the gap size as small as 1 nm via atomic layer deposition (ALD) of gap-filling insulators^34^. The origin of strong optical resonances in a coaxial aperture was previously explained using a mechanism based on the zeroth-order Fabry-Pérot (FP) resonance of the gap mode^31,34^. Alternatively, this mode can be interpreted as the effective epsilon-near-zero (ENZ) phenomenon. ENZ photonics has provided a convenient framework to describe a wide range of phenomena such as electromagnetic tunneling through ultranarrow channels operating at the cutoff condition, uniform phase accumulation, large field enhancement, supercoupling, optical nonlinearity, and nonlocality^35–38^. Unlike the nonlocality observed in other plasmonic modes with a large wavevector in the propagation direction, the cutoff ENZ mode in a coaxial aperture has a vanishingly small wavevector component along the propagation axis, yet exhibits strong nonlocality as shown below.

Coupling Maxwell’s equations leads to the wave equation in momentum and frequency space:

2 $${\mathbf{k}} \times \left[ {{\mathbf{k}} \times {\mathbf{E}}\left( {k,\omega } \right)} \right] = {\mathbf{k}}\left( {{\mathbf{k}} \cdot {\mathbf{E}}} \right) - k^2{\mathbf{E}} = - \varepsilon \left( {k,\omega } \right)\frac{{\omega ^2}}{{c^2}}{\mathbf{E}}.$$

Equation (2) has two solutions depending on the polarization of the electric field. For transverse waves, the divergence-free solution ${\mathbf{k}} \cdot {\mathbf{E}} = 0$ yields the usual dispersion relation of $k^2 = \varepsilon \left( {k,\omega } \right)\omega ^2/c^2$. On the other hand, the curl-free solution ${\mathbf{k}} \times {\mathbf{E}} = 0$ is satisfied by longitudinal waves $({\mathbf{k}}\parallel {\mathbf{E}})$, when the condition $\varepsilon \left( {k,\omega } \right) = 0$ is fulfilled. In order to find the longitudinal solutions that satisfy Eq. (2), we need an explicit expression for *ε*(*k*,*ω*) = 0. For a metal, the electron dynamics can be described, in principle, by the Lindhard function^39^, which takes into account the full quantum nature of the electron gas. In general, however, when dealing with inhomogeneous, finite size systems, it becomes very difficult to work in the reciprocal *k*-space and a real-space implementation is required. To this end, a hydrodynamic description of the electron dynamics inside a metal provides a relatively simple tool to predict nonlocal response effects in large plasmonic systems. The hydrodynamic model can be summarized by the following equation:^40^

3 $$\beta ^2\nabla \left( {\nabla \cdot {\mathbf{J}}} \right) + \left( {\omega ^2 + i\gamma \omega } \right){\mathbf{J}} = i\omega \varepsilon _0\omega _{\mathrm{p}}^2{\mathbf{E}}.$$

The first term in Eq. (3) gives rise to the nonlocal response—the electric field at the point **r** not only generates currents at **r** but also in its neighborhood—due to the presence of spatial derivatives. From a physical point of view, the first term in Eq. (3) arises from the electron quantum pressure that prevents charges in the metal to occupy the exact same state, i.e., induced charges do not collapse into a delta function at the metal surface. The nonlocal parameter *β* is proportional to the Fermi velocity *v*_F_, and for a 3-D system the Thomas–Fermi approximation gives $\beta ^2 = v_{\mathrm{F}}^2/3$. At high frequencies (including optical frequencies), however, $\beta ^2 = 3v_{\mathrm{F}}^2/5$, such that Eq. (3) gives the same result in a free-electron gas as the Lindhard function, up to $O(k^2)$^41^.

It is useful to extract from Eq. (3) the spatially dispersive permittivity *ε*_L_ for the longitudinal modes:

4 $$\varepsilon _{\mathrm{L}}\left( {k,\omega } \right) = 1 - \frac{{\omega _{\mathrm{p}}^2}}{{\omega ^2 + i\gamma \omega - \beta ^2k^2}}.$$

We notice (neglecting for a moment the damping term) that the dispersion relation of longitudinal waves *ε*(*k*,*ω*) = 0 is satisfied by Eq. (4) for real *k* only if *ω* > *ω*_P_, giving rise to propagating bulk plasmons. Because of inter-band absorption, it is hard to observe these waves in real metals, although some resonances due to bulk plasmons can be detected in sufficiently small systems^42^. For *ω* < *ω*_P_, however, solutions with imaginary *k* exist. These solutions are associated with evanescent bulk plasmons. Although they do not propagate in the bulk region, they nonetheless exist at the metal surface and are responsible for inducing a charge accumulation (or depletion) at the metal surface that spreads to the internal metal volume, causing an observable deviation of optical properties compared with a purely local description. In the Thomas–Fermi approximation, the hydrodynamic model does not account for electron spill-out nor electron tunneling. These effects only become important for gaps below half a nanometer, and can therefore be safely neglected in this work. In general, however, they can be accounted for by including gradient dependent corrections to the kinetic energy functional of the electron gas^41,43,44^.

Nonlocal effects have a strong impact on systems supporting ENZ modes, such as nanowire-based metamaterials^45^ and thin films^42^, for which the ENZ condition occurs around their plasma frequencies (*ω*_P_). The coaxial nanoaperture can serve as a practical platform to study the nonlocality triggered by the ENZ effect in the long-wavelength regime, since its effective ENZ mode can be widely tuned from the near- to mid-IR, and even terahertz regimes while confining the field inside sub-10-nm gaps^34,46,47^. In our previous works in mid-IR and terahertz frequencies^34,46,47^, the EOT phenomena were demonstrated through such ultranarrow coaxial nanoapertures. While the ENZ mode resonances at 10 and 7 nm gaps were in good agreement with the local modeling results, the blueshifts of the ENZ mode resonance from the local modeling results began to appear as the gap size decreased below 5 nm and became apparent at 2 nm gap. Such a large deviation, which cannot be explained by only the fabrication imperfection or variation, drives us to elucidate the origin of the discrepancy between the experiment and the local calculation occurring in ultranarrow gaps in the perspective of nonlocality by combining the sophisticated fabrication process and the advanced HDG modeling equipped with a hydrodynamic model.

**Fig. 1:**
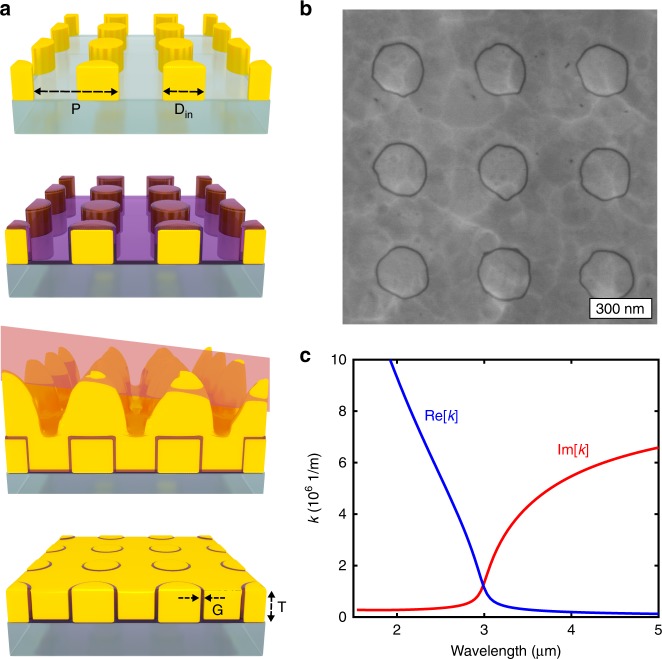
Schematics of the coaxial nanoapertures and its epsilon-near-zero (ENZ) optical property. **a** The illustration of coaxial nanoapertures shows geometrical parameters including an inner diameter (*D*_in_), gap size (*G*), period (*P*), and thickness of the gold film (*T*). Processing scheme: (1) Gold nanopillars are patterned via electron-beam lithography; (2) ALD coating of gap-filling insulators (Al_2_O_3_; thickness from 1 to 10 nm); (3) gold sputtering; (4) glancing-angle ion milling to planarize the top surface and expose the Al_2_O_3_-filled nanocoax array. **b** Scanning electron microscopy (SEM) images of coaxial nanoapertures with a 10 nm alumina gap, 250 nm diameter, 500 nm period, and 150 nm Au thickness. **c** Dispersion of a guided mode (real and imaginary parts) of a coaxial waveguide (250 nm diameter and 10 nm gap width) in a gold film illuminated with a linearly polarized beam at normal incidence

As illustrated in Fig. 1a, our device consists of an array of coaxial nanoapertures arranged in a square lattice. After patterning gold pillars (250 nm diameter) on a sapphire wafer, ALD-grown Al_2_O_3_ films on the sidewalls precisely control the gap size. After depositing a second layer of gold film and glancing-angle ion milling, the planarized top surface exposes a dense array of coaxial nanoapertures. The diameter of each coax (250 nm) and the array periodicity (500 nm) are about an order of magnitude smaller than the MIR resonance wavelength (~3–7 µm), thus our structure can be considered a metamaterial. Each coaxial aperture can support a TE_11_-guided mode at the cutoff frequency, when illuminated with linearly polarized light at normal incidence. The fundamental TEM mode of the coax, however, cannot be excited in that configuration due to the mismatch in mode symmetry. The cutoff resonance is entirely determined by the geometry of a single coaxial waveguide such as the inner diameter (*D*_in_) and gap size (*G*) following the dispersion relation in the coaxial waveguide derived below. Let us start from the dispersion relation of planar metal–insulator–metal (MIM) structures:

5 $$\tanh \left( {k_{\mathrm{i}}\frac{d}{2}} \right) = - \frac{{k_{\mathrm{m}}\varepsilon _{\mathrm{i}}}}{{k_{\mathrm{i}}\varepsilon _{\mathrm{m}}}}$$

where $k_{\mathrm{m}}^2 = \kappa _{{\mathrm{mim}}}^2 - k_0^2\varepsilon _{\mathrm{m}}$ and $k_{\mathrm{i}}^2 = \kappa _{{\mathrm{mim}}}^2 - k_0^2\varepsilon _{\mathrm{i}}$. Here *ε*_m_ and *ε*_i_ are the relative permittivities of the metal and the insulator, respectively, *k*_0_ = *ω*/*c* is the free-space wavenumber, and *d* is the space between two metal plates. Equation (5) can also approximately describe the dispersion relation of a coaxial waveguide by inserting the total propagation number, *κ*_mim_, consisting of two components^48^:

6 $$\kappa _{{\mathrm{mim}}}^2 = \kappa ^2 + k_\theta ^2,\quad k_\theta 2\pi r = 2\pi \Gamma$$

where *κ* is the wavevector component along the propagation axis and *k*_*θ*_ is transverse component in the cross-sectional plane, *r* is the radius of the coaxial waveguide, and Γ is an integer representing the angular momentum. Combining Eqs. (5) and (6) leads to the dispersion relation for a coaxial waveguide depicted in Fig. 1c. The real part of the propagating wavenumber *κ* vanishes spectrally close to the cutoff frequency, whereas its imaginary part increases over the cutoff frequency. This points out that the effective permittivity of a coaxial waveguide at the cutoff frequency is near-zero so that the system behaves as if it is filled with a near-zero permittivity metamaterial, thus showing effective ENZ properties.

Although in this work we perform full-wave 3-D numerical calculations of the coaxial waveguide system, the dispersion relation obtained above can be used to intuitively analyze the nonlocal effect at the ENZ cutoff frequency. In the coaxial waveguide, the perpendicular wavevector component *k*_m_ that determines the exponential decay into the metal surface can be derived as $k_{\mathrm{m}} = \sqrt {\kappa ^2 + \left( {\frac{1}{r}} \right)^2 - k_0^2\varepsilon _{\mathrm{m}}}$. As expected, as the radius *r* becomes infinitely large, the coax structures tend toward a planar MIM structure, so that the geometric term disappears and the in-plane wavevector component *κ* becomes a dominant parameter to the nonlocal effect. Conversely, *k*_m_ in the coaxial waveguide is largely affected by the geometric term *r*, since *κ* is very small for the ENZ mode. Thus, as the radius is reduced, *k*_m_ can become very large, boosting the nonlocal effect at the ENZ cutoff condition in the coaxial aperture. However, it is interesting to note that 1/*r* is just the transverse component of the coaxial wavevector, *k*_*θ*_ = Γ/*r*. It is clear from the first of Eqs. (6) that for $\kappa \cong 0,\kappa _{{\mathrm{mim}}} \cong k_\theta ,$ so that for a coaxial waveguide, *k*_*θ*_ plays the same role as *κ*_mim_ in the MIM system. The main difference is that while the light is “going around” the coax ring, it is also slowly moving (*v*_g_ = δ*ω*/δ*k*) forward through the film, maximizing the interaction time of the light with the metal, and thus increasing the nonlocal signature of the system.

### 3-D computational modeling of mid-IR nonlocality

**Fig. 2:**
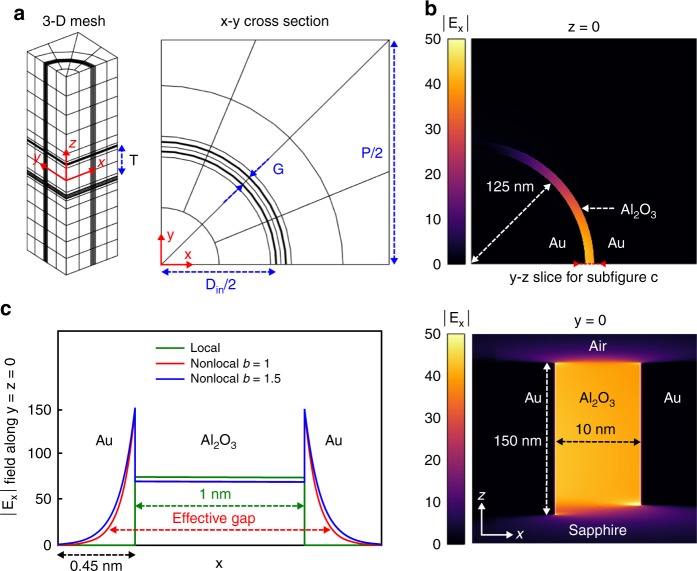
Computational modeling of nonlocality in a coaxial nanoaperture. **a** 3-D mesh used for simulations consisting of 1960 hexahedra, with a *z*-constant cross section. Curved geometries are approximated with a cubic representation. Axes are provided for reference. **b** Magnitude of the field enhancement on two cross sections for the 10 nm gap structure illuminated at resonant wavelength 2.87 µm, computed with the nonlocal model (*b* = 1). Plasmonic hotspots appear along *y* = 0 near the upper and lower metal tips. **c** Magnitude of enhancement computed along the *x* axis for the 1 nm gap and all the models, at the resonant wavelength for each case. Enhancement in the metal vanishes for the local model, whereas for the nonlocal model enhancement decays a skin depth proportional to the nonlocal parameter *β*. Skin depth does not depend on gap size, thus narrower gaps exhibit more prominent relative gap enlargement

While the above analytical approach—based on the assumption that the material parameters vary slowly with respect to the wavelength—can provide intuitive ideas of how the ENZ mode should behave in coaxial apertures, full-wave 3-D simulations are required to quantitatively analyze the resonance patterns of the coaxial structure and compare with experimental data. For this purpose, we have addressed its simulation using the hybridizable discontinuous Galerkin method. The HDG method is a high-order accurate, stabilized and locally conservative finite-element numerical scheme designed to resolve rapid field variations in complex geometries spanning multiple length scales. This method has allowed researchers to solve acoustic, elastic, and electromagnetic wave propagation problems more efficiently than other presently available finite-element techniques. The HDG method for 3-D time-harmonic Maxwell’s equations^49^ has been used for local nanophotonics simulations^34^ showing a remarkable agreement with experimental results. The HDG method has been extended to account for nonlocality in 3-D structures^50^, more specifically a periodic nanoaperture structure for low terahertz frequencies. Unlike other commercially available finite-different time-domain (FDTD) or finite-element method (FEM) solvers, the HDG method employs arbitrarily high-order approximations, thereby reducing the numerical error to levels where the solution is practically insensitive to the fineness of the mesh discretization. High accuracy and stability are critically important when one considers resonant phenomena and solutions which exhibit extreme-scale variations and highly localized features such as ultrathin charge distribution layers resulting from nonlocality. In addition, the HDG formulation can easily accommodate divergence-free constraints without resorting to curl-conforming subspaces and exhibits less globally coupled unknowns and higher convergence rates than other finite-element methods, hence is more computationally efficient and accurate, see Supplementary Note 1. In this paper, we use the novel HDG method for the hydrodynamic model introduced in ref. ^50^ to simulate nonlocality on ultranarrow coaxial structures. To the best of our knowledge, this is the first time that full 3-D nonlocal plasmonic simulations have been performed for extended 3-D periodic nanostructures on the long-wavelength MIR regime. The simulations are performed on a highly anisotropic mesh with 1960 hexahedra, with a higher concentration of elements near the metal-insulator boundaries of the structure, see Fig. 2a and the Methods section for further details. For the different gap widths, we compute the LRA, as well as the nonlocal response for $\beta ^2 = b\beta _0^2$, where the baseline value for the nonlocal parameter is the first-principle high-frequency regime value, $\beta _0^2 = 3v_{\mathrm{F}}^2/5$, and *b* is the only fitting parameter in our model (note the LRA would correspond to *b* = 0). For the simulations we first considered *b* = 1, and in light of the significant blue-shift observed with respect to experiments for small gaps (6.6% for 1 nm and 7.5% for 2 nm), we increased *b* to better match the measured resonances. The best agreement with experimental data was obtained when *b* = 1.5.

For *G* = 10 nm and *b* = 1, we show in Fig. 2b the magnitude of the field enhancement $\left| {E_x} \right|$ of the ENZ mode for the resonant wavelength 2.87 μm. The plasmonic hotspots are located near the metal tips, and the mode is nearly constant along the thickness of the gap. The enhancement is maximized in the direction of the polarization. An important aspect of nonlocal simulations is that, contrary to LRA, the electric field penetration inside the metal can be captured. The one-dimensional $\left| {E_x} \right|$ profile along *y* = *z* = 0 for LRA and the nonlocal response is depicted in Fig. 2c, at the resonant wavelength of each model for the 1 nm gap. The nonlocal model exhibits lower enhancement in the alumina, and inside the metal the enhancement decays more gradually than with LRA, forming a boundary-layer type structure with a skin depth that depends only on the nonlocal parameter *β*. Hence, the gap seen by the incident wave is effectively enlarged in the nonlocal model. The relative effective enlargement is more significant for narrower apertures, since the skin depth is constant across gap widths.

**Fig. 3:**
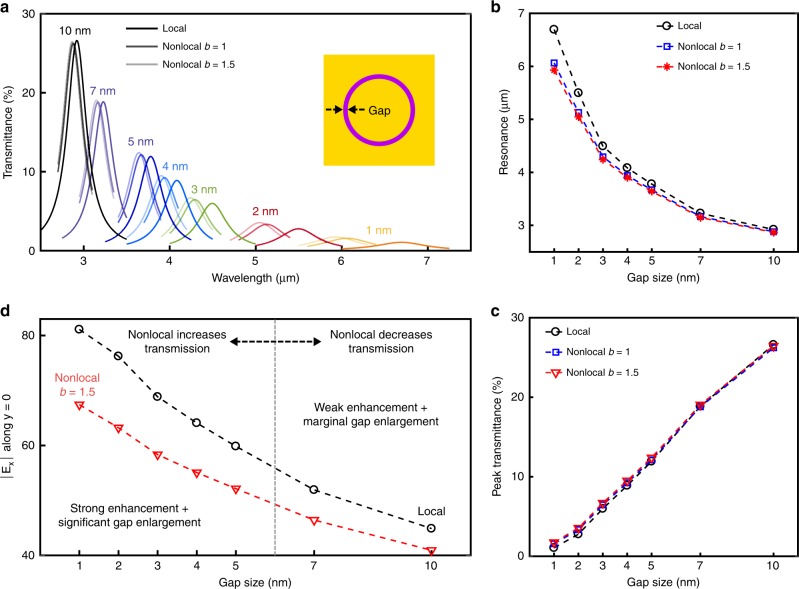
Simulation results for the coaxial nanoaperture. **a** Transmission spectra for all gap widths and models. **b**, **c** The resonant wavelength and peak transmittance are tracked for the different models and depicted as a function of gap size. Nonlocality always blueshifts resonances, whereas it increases transmission only for sub-7 nm gaps. **d** Field enhancement integrated along the alumina at *y* = 0 vs gap width. Nonlocality decreases enhancement, and for large gaps the wider effective gap does not compensate the decay in enhancement, thus transmission decreases. For small gaps, the larger effective gap outweighs the decrease in enhancement, hence transmission increases. The breakpoint occurs around 7 nm

The transmittance spectra for all gaps and models, the tracking of the resonant wavelength and peak transmittance are shown in Fig. 3a–c. The nonlocal model blueshifts the resonances with respect to LRA, and larger relative shifts are observed for larger *b* values (1 nm gap: 9.5% for *b* = 1 and 11.5% for *b* = 1.5) as well as for smaller gaps (9.5% for 1 nm gap and 1.4% for 10 nm gap). An interesting phenomenon we noticed is that nonlocal transmittance is greater than local transmittance only for gaps below 7 nm. Indeed, in addition to enlarging the aperture (increase in transmittance), nonlocality reduces peak field enhancement (decrease in transmittance). For small gaps, we observe an overall increase in transmittance since the relative gap enlargement is significant (skin depth depends only on *β*) and absolute enhancement is still high, albeit lower than with LRA, see Fig. 3d. However, for large gaps the aperture enlargement is not sufficient to compensate for the quenching of enhancement, hence transmittance decreases.

### Experimental verification of mid-IR nonlocality

**Fig. 4:**
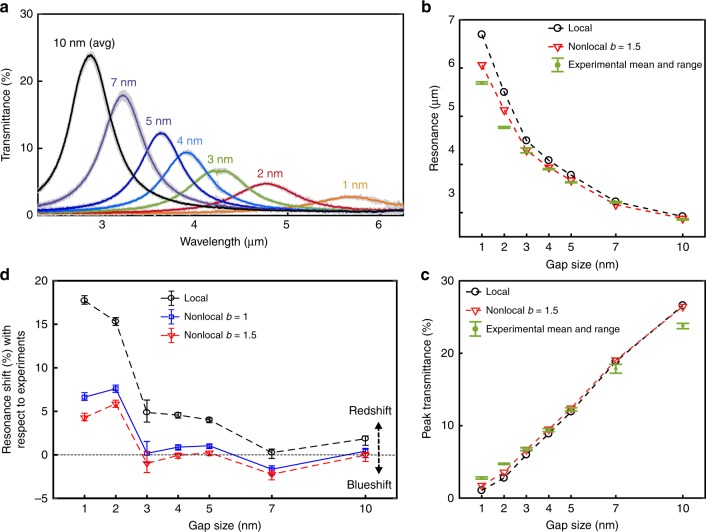
Comparison of experimental data with modeling results. **a** Transmission spectra measured from ENZ coaxial metamaterials with different gap sizes. Gray scattering points indicate the distribution of spectra measured from eight different locations in each sample. Solid line is the average of eight measured spectra. **b**, **c** The resonance wavelengths and peak transmittance are plotted as a function of gap size for the comparison of an experiment with local and nonlocal modeling, respectively. Experimental data have an error margin calculated from the distribution of eight measured spectra. **d** Relative resonance shifts are calculated from (**b**) and then plotted as a function of gap size

Once the inner diameter of a coaxial aperture (*D*_in_) is fixed, the resonance of the ENZ mode can be tuned by changing its gap size, which is defined by the thickness of the alumina ALD layer in our fabrication process. It provides us with the degree of freedom necessary to explore the nonlocal effect on the spectral resonance shift as well as the transmitted intensity at the ENZ mode by scaling the thickness of the alumina layer down from 10 to 1 nm. We measured mid-IR transmission through large-area coaxial aperture arrays with seven different gap sizes (10, 7, 5, 4, 3, 2, 1 nm) as shown in Fig. 4a, and compared them with numerically calculated results. The transmission spectrum of each sample was measured at eight different positions on the sample to obtain a statistically valid data set incorporating the inhomogeneity of coaxial apertures. Gray scattered points indicate the distribution of the measured data set, and solid line represents the average of eight data points. As seen in Fig. 4, the ENZ mode of a 10 nm gap sample shows a very strong resonance peak (as high as 24% in absolute transmission) through a very small open area of 3%. As the gap size is reduced, the ENZ-mode resonance shifts toward longer wavelengths. The resonance as a function of the gap size is plotted in Fig. 4b and compared with the local and nonlocal modeling results. It is clearly seen that the deviation of experimentally measured resonance from the local modeling results tends to augment as the gap shrinks. The experiment and local modeling results agree well for 10 and 7 nm gaps. Below 7 nm gap size, however, the discrepancy between measurements and the local model calculations begins to increase. In the extreme case, the measured resonance of a 1 nm gap sample is 1000 nm away from what the local model predicts. On the other hand, the experimental and nonlocal modeling (*b* = 1.5) results match well down to the 3 nm gap size, while they still show ~5% deviation for 1 and 2 nm gap sizes (see Fig. 4d).

In addition to the influence of nonlocality on the resonance shift, we investigate how it affects the MIR transmission intensity through ultranarrow coaxial apertures. The transmission measurements are compared with modeling results in Fig. 4c. For gap sizes of 10, 7, 5, and 4 nm, we observe a progressive improvement of the agreement between the calculated intensity values and the measured transmittance as the gap size shrinks. Although the measured quantitative values could be affected by many parameters, the qualitative trend that we observe could be explained by the following theoretical argument. The broadening of the transmittance resonances (hence their peak intensity values) can be associated with two different mechanisms: on the one hand, there are fabrication imperfections (i.e., shape and size of the rings are not perfectly uniform) that should introduce a constant broadening across the different gap sizes; on the other hand, there is the intrinsic broadening due to ohmic losses in the metal, which increases for smaller gaps due to the fact that the gap-plasmon mode becomes more lossy. Since in our numerical calculation we assume perfectly uniform structures, agreement with experimental data improve as the ohmic losses increase, dominating the overall broadening process. For sub-3 nm gap sizes, however, the measured transmission is higher than predicted by the simulations. In particular, at the 1 nm gap size, the measured intensity is two times larger than the calculated value. Even after incorporating the nonlocal effect in modeling, large deviations of resonance shift as well as transmission intensity from the nonlocal modeling are still observed at extremely small gap dimensions of 1 and 2 nm. As illustrated in Figs. 2 and 3, the nonlocality and the resulting smearing of electrons can effectively enlarge the gap size that contributes to the outgoing radiation from the aperture. This enlargement of the effective gap size will be more prominent for smaller gap sizes. However, we assumed a constant nonlocal parameter (*b*) for all gap sizes from 10 to 1 nm, which may give rise to the deviation from the experimental results in terms of resonance shift and enhanced transmission.

## Discussion

Our study clearly shows that the nonlocal effect cannot be ignored for accurate theoretical prediction and experimental characterization of ultranarrow gap structures in the MIR domain. In particular, the coax geometry proposed and the fabrication techniques used minimize the impact of roughness on the system resonances. In fact, because the ALD process is highly conformal, the presence of roughness would randomly shift the resonance of each aperture toward higher or lower frequencies, producing a global broadening of the resonance without affecting the peak center of mass. Our observations confirm a trend that is accurately reproduced by numerical calculations, leaving in our opinion, a small margin to other interpretations. These results are consistent with previously published work^12^ and further corroborate the fact that nonlocality (or nonlocal-like effects) leads to the blue-shift of gap-plasmon resonances in gold nanostructures with respect to local predictions. Our approach based on the hydrodynamic model and full 3-D HDG simulations provides a practical route and unparalleled efficiency for researchers to design high-performance MIR nanogap antennas, large-area ENZ metamaterials, and biochemical sensors^51,52^ with nonlocal corrections. Our experiments show that as the gap size shrinks below 1 nm the physics might actually become more complex than what is captured by the hydrodynamic description. In the future, effects such as light-induced electron tunneling, electron spill-out and other quantum phenomena should be included in addition to nonlocality. These effects will pose significant new challenges from a computational perspective, as additional nonlinear equations that describe the behavior of electron density need to be simultaneously solved, and extremely refined meshes are required to properly capture the quantum phenomena. Furthermore, the thickness dependence of the dielectric spacer might play a crucial role in describing the electromagnetic response of sub-nanometer-gap systems. While daunting challenges exist for simulating large 3-D quantum plasmonic devices, the HDG method combined with the ever-increasing computational and storage power will provide researchers with a new route to simulate complex geometries involving extreme-scale size mismatches and rapid variations in atomic-scale charge distribution layers. On the experimental side, atomic layer lithography will also enable researchers to overcome technological challenges of mass-producing ultrasmall resonant gaps as well as probing and harnessing quantum plasmonic phenomena at the wafer scale.

## Methods

### Sample fabrication via atomic layer lithography

A sapphire wafer (University wafer) was spin-coated with poly(methyl methacrylate) resist (MicroChem, 950 PMMA C4) at 3000 r.p.m. for 60 s, followed by thermal curing on a hot plate at 180 °C for 15 min. After a 20-nm-thick Au layer was sputtered on the electron-beam resist to prevent electrons from piling up, a nanohole array (250 nm diameter and 500 nm period) was patterned via electron-beam lithography (VISTEC, EBPG5000+) at 100 KeV beam energy and 1000 μC/cm^2^ exposure dose. After removal of Au in gold etchant (Sigma-Aldrich) and developing with a solution of MIBK:IPA (1:3) for 90 s, 3-nm Ti and 200-nm Au film were then directionally deposited on the patterned substrate using electron-beam evaporator (CHA, SEC 600). After a lift-off process in acetone and an oxygen plasma cleaning (STS, 320PC) at 100 W for 30 s to remove resist residue, the resultant Au nanodisk array was coated with an Al_2_O_3_ film using ALD (Cambridge Nano Tech Inc., Savannah) at a typical deposition rate of 1 Å per cycle at 250 °C, followed by conformal deposition of a 400 nm Au film using an electron-beam evaporator (CHA, SEC 600) with a planetary fixture. Finally, the structures were planarized via ion milling (Intlvac, Nanoquest) with an Ar beam of 130 mA and 36 V incident at 10º from the horizontal plane.

### FTIR measurement

Transmission spectrum measurement was performed on a Thermo Fisher Scientific Nicolet iS 50 FTIR spectrometer with a liquid nitrogen-cooled mercury cadmium telluride (MCT) detector under the acquisition settings of 150 scans with 4 cm^−1^ resolution. All samples were measured using a Nicolet continuum infrared microscope with the Reflechromat objective and condenser of 15× and a 0.58 numerical aperture. The signals were collected from the sample area of 100 × 100 μm^2^ through a knife edge aperture. The background signal was measured from a bare sapphire substrate under identical acquisition conditions and was then used for the normalization.

### Computer simulations

The results in Figs. 2 and 3 have been obtained by simulating the 3-D time-harmonic Maxwell’s equations with both the LRA and the hydrodynamic model for the metal, using the HDG method. The 3-D mesh is formed by extruding a 2-D cubic mesh that is highly anisotropic to capture the extreme features that develop at the metal-alumina interface. The values for the inner diameter and gap width used for the simulations correspond to statistical averages obtained from the fabricated devices, see Supplementary Note 3. For each gap size we use distinct nodal points, but mesh connectivities are identical across gap sizes. To reduce computational cost, the symmetries of the lattice are capitalized upon, thus only one quarter of the unit cell is simulated. The structure is illuminated from below with an *x*-polarized plane wave, hence PEC conditions are prescribed on the *x*-constant boundaries, PMC conditions on the *y*-constant boundaries and first-order absorbing conditions on the top and bottom boundaries. The total height of the mesh is 1 µm. Absorbing conditions are validated by simulating the structure with PML layers on the outer boundaries. For the 1 nm gap mesh, the volume ratio between the largest and the smallest element is 10^5^. The mesh resolution is chosen so that transmittance results are grid-converged to <0.1% at resonance. All relevant formulation and implementation details of the HDG method for these nanocoax simulations may be found in Supplementary Note 1. The dielectric constant for thin-film alumina is extracted from measurements in Kischkat^53^, whereas the optical constants for gold are obtained from Olmon et al.^54^.

## Supplementary information

Supplementary Information

## Data Availability

The data that supports the finding of this study are available from the corresponding author upon request.

## Code availability

The code used to compute the results in this paper may be downloaded from https://github.com/ferranvidal/nanocoax.
