# Supplementary material for: Modeling and observation of mid-infrared nonlocality in effective epsilon-near-zero ultranarrow coaxial apertures
Source: Nat Commun. 2019 Oct 2;10:4476. doi: 10.1038/s41467-019-12038-3 (PMC6775091; doi:10.1038/s41467-019-12038-3)
Supplement: Supplementary file 1 — Supplementary Information [file 41467_2019_12038_MOESM1_ESM.pdf]

Supplementary Information:

Modeling and Observation of Mid-Infrared Nonlocality in Effective Epsilon-Near-Zero Ultranarrow Coaxial Apertures

Yoo *et al.*

Supplementary Note 1: Hybridizable Discontinuous Galerkin method

Governing equations

The hybridizable discontinuous Galerkin (HDG) method [49,50] is an advanced numerical scheme that is unstructured, high-order accurate, locally conservative, accurately approximates extreme-size scale mismatches, enables a natural treatment of both boundary conditions and material contrasts at interfaces. In addition, the HDG is efficient since it gives rise to a linear system whose degrees of freedom are defined only on the faces of the discretization cells. Hence, it is well suited to numerically simulate wave propagation problems.

In this section, we describe the equations and implementation used in the simulation of the nanophotonic structure shown in Figure 1. The HDG method was used to simulate the full 3-D electromagnetic response on a given computational domain $\Omega \subset \mathbb{R}^3$ that contains the nanophotonic structure. The domain Ω can be split according to whether the material is a dielectric Ω_d or a metal Ω_m, thus $\Omega = \Omega_\mathrm{d} \cup \Omega_\mathrm{m}$, with boundary $\partial\Omega = \Gamma$. In Ω_d the scattering is governed by the time-harmonic Maxwell's equations for a single frequency ω

$$\nabla \times \mathbf{E} - i\omega\mathbf{H} = 0, \qquad \nabla \times \mathbf{H} + i\omega\varepsilon_\mathrm{d}\mathbf{E} = 0, \tag{1}$$

where ε_d is the relative permittivity of the dielectric. For the simulations the permittivity of the sapphire substrate is given by the Sellmeier's formula

$$\varepsilon_\mathrm{d} = 1 + \frac{1.4313493\lambda^2}{\lambda^2 - 0.0726631^2} + \frac{0.65054713\lambda^2}{\lambda^2 - 0.1193242^2} + \frac{5.3414021\lambda^2}{\lambda^2 - 18.028251^2}\,, \tag{2}$$

for λ in micron, whereas the dielectric constant for Al_2O_3 is extracted from the experimental measurements reported in [53].

In the metal Ω_m, the time-harmonic Maxwell's equations are augmented with the hydrodynamic model for metals, namely

$$\begin{aligned} \nabla \times \mathbf{E} - i\omega\mathbf{H} &= \mathbf{0}, \\ \nabla \times \mathbf{H} + i\omega\varepsilon_\infty\mathbf{E} &= \mathbf{J}, \\ \beta^2\nabla\rho + (\gamma - i\omega)\mathbf{J} &= \omega_\mathrm{p}^2\mathbf{E}, \\ \nabla\cdot\mathbf{J} &= i\omega\rho, \end{aligned} \tag{3}$$

where $\mathbf{J}$ is the internal current and ρ the internal charge density. Parameters (ε_∞, ω_p, γ, β) represent the metal permittivity, plasma frequency, the electron collision rate and the nonlocal parameter respectively. We use $\varepsilon_\infty = 1$, $\hbar\omega_\mathrm{p} = 8.45$ eV, $\hbar\gamma = 0.047$ eV [54], and for

the nonlocal parameter we have $\beta^2 = b\,3/5v_{\mathrm{F}}^2$, with $v_F = 1.39 \cdot 10^6$ m/s being the Fermi velocity of gold and b a fitting parameter to regulate the "amount" of nonlocality.

In order to alleviate the computational cost, we reduce the computational domain Ω to be one quarter of the unit cell, and capitalize the symmetries and periodicity of the structure. The mesh used in the simulations is shown in Fig 2a. For an x-polarized incident plane wave $(\mathbf{E}_0, \mathbf{H}_0)$ propagating upwards, that is $\mathbf{E}_0 = \exp(i\omega\sqrt{\varepsilon_{\mathrm{d}}}z)\hat{\boldsymbol{x}}$, periodicity is imposed by prescribing $\mathbf{E} \times \mathbf{n} = 0$ at the x-constant boundaries Γ_x and $\mathbf{H} \times \mathbf{n} = 0$ at the y-constant boundaries Γ_y. The system above is complemented with first-order absorbing boundary conditions for the top and bottom boundaries, also known as radiation boundaries Γ_{rad}

$$\mathbf{H} \times \mathbf{n} - \sqrt{\varepsilon_{\mathrm{d}}}\,\mathbf{n} \times \mathbf{E} \times \mathbf{n} = \mathbf{H}_0 \times \mathbf{n} - \sqrt{\varepsilon_{\mathrm{d}}}\,\mathbf{n} \times \mathbf{E}_0 \times \mathbf{n}\,. \tag{4}$$

The right-hand-side of the above equation represents the incoming flux, and is hereafter referred to as $\mathbf{f}_0$. Since the radiation boundaries are placed far away from the device, they belong to Ω_{d}. Radiation boundaries ensure the outgoing waves are absorbed, and hence do not propagate back into the computational domain. For the metal-dielectric interfaces, we need an additional no-spill out condition, which enforces the electrons to remain inside the metal, given by $\mathbf{J} \cdot \mathbf{n} = 0$. Finally, symmetries need to be imposed in the charge density distribution, which for an x-polarized wave read $\rho = 0$ at $\Gamma_x \cap \partial\Omega_{\mathrm{m}}$ and $\mathbf{J} \cdot \mathbf{n} = 0$ at $\Gamma_y \cap \partial\Omega_{\mathrm{m}}$.

Transmittance is computed as the ratio between the transmitted power across a z-constant surface A above the gold layer and the incident power across a z-constant surface A_0 below the gold layer, namely

$$\frac{\int_A |\Re\left[\mathbf{E} \times \mathbf{H}^*\right] \cdot \mathbf{n}|}{\int_{A_0} |\Re\left[\mathbf{E}_0 \times \mathbf{H}_0^*\right] \cdot \mathbf{n}|}\,. \tag{5}$$

The enhancement on a surface A similarly computed as the following ratio

$$\frac{\int_A |\mathbf{E} \cdot \mathbf{p}|}{\int_A |\mathbf{E}_0 \cdot \mathbf{p}|}\,, \tag{6}$$

where $\mathbf{p}$ is the polarization vector.

Implementation

We denote by $\mathcal{T} = \mathcal{T}_{\mathrm{d}} \cup \mathcal{T}_{\mathrm{m}}$ a triangulation of disjoint regular elements T that partition Ω. The set of element boundaries is then defined as $\partial\mathcal{T} := \{\partial T : T \in \mathcal{T}\}$. For an arbitrary element $T \in \mathcal{T}$, $F = T \cap \Gamma$ is a boundary face, and for arbitrary $T_{\mathrm{m}} \in \mathcal{T}_{\mathrm{m}}$ and $T_d \in \mathcal{T}_d$, $F = T_{\mathrm{m}} \cap T_d$ is a metal-dielectric interface, provided they have nonzero 2D Lebesgue measure. We finally denote by $\mathcal{F}^o$, $\mathcal{F}^\partial$ (for both metal and dielectric) and $\mathcal{F}^i$

the set of interior, boundary and interface faces respectively, and the total set of faces $\mathcal{F} = \mathcal{F}_d^o \cup \mathcal{F}_d^\partial \cup \mathcal{F}_{\mathrm{m}}^o \cup \mathcal{F}_{\mathrm{m}}^\partial \cup \mathcal{F}^i$.

Let $\mathcal{P}^p(\Omega)$ denote the space of complex-valued polynomials of degree at most p on $\Omega = \Omega_{\mathrm{d}} \cup \Omega_{\mathrm{m}}$. We introduce the following approximation spaces

$$\begin{aligned} W_h &= \{w \in L^2(\Omega) : w|_T \in \mathcal{P}^p(T),\ \forall T \in \mathcal{T}_h\}, \\ \boldsymbol{W}_h &= \{\boldsymbol{\xi} \in \boldsymbol{L}^2(\Omega) : \boldsymbol{\xi}|_T \in [\mathcal{P}^p(T)]^3,\ \forall T \in \mathcal{T}_h\}, \\ M_h &= \{\mu \in L^2(\mathcal{F}) : \mu|_F \in \mathcal{P}^p(F),\ \forall F \in \mathcal{F}\}, \\ \boldsymbol{M}_h &= \{\boldsymbol{\mu} \in \boldsymbol{L}^2(\mathcal{F}) : \boldsymbol{\mu}|_F \in \mathcal{P}^p(F)\mathbf{t}_1 \oplus \mathcal{P}^p(F)\mathbf{t}_2,\ \forall F \in \mathcal{F}\}, \end{aligned} \tag{7}$$

where $\mathbf{t}_1$, $\mathbf{t}_2$ are linearly independent vectors tangent to the face. Boundary conditions are included by setting $\boldsymbol{M}_h(0) = \{\boldsymbol{\mu} \in \boldsymbol{M}_h : \mathbf{n} \times \boldsymbol{\mu} = 0 \text{ on } \partial\Omega\}$ and $M_h(0) = \{\mu \in M_h : \mu = 0 \text{ on } \partial\Omega\}$, where $\mathbf{n}$ is the outward-pointing normal to the face.

Finally, we define the various Hermitian products for the above finite element spaces. The volume inner products are defined as

$$(\eta, \zeta)_{\mathcal{T}} := \sum_{T \in \mathcal{T}} (\eta, \zeta)_T, \qquad (\boldsymbol{\eta}, \boldsymbol{\zeta})_{\mathcal{T}} := \sum_{i=1}^{3} (\eta_i, \zeta_i)_{\mathcal{T}}, \tag{8}$$

and the surface inner products by

$$\langle \eta, \zeta \rangle_{\partial\mathcal{T}} := \sum_{T \in \mathcal{T}} \langle \eta, \zeta \rangle_{\partial T}, \qquad \langle \boldsymbol{\eta}, \boldsymbol{\zeta} \rangle_{\partial\mathcal{T}} := \sum_{i=1}^{3} \langle \boldsymbol{\eta}_i, \boldsymbol{\zeta}_i \rangle_{\partial\mathcal{T}}. \tag{9}$$

For two arbitrary scalar functions η and ζ, its scalar product $(\eta, \zeta)_\Omega$ is the integral of $\eta\zeta^*$ on Ω.

The HDG method seeks to approximate $(\mathbf{E}, \mathbf{H}, \mathbf{J}, \rho)$ with $(\mathbf{E}_h, \mathbf{H}_h, \mathbf{J}_h, \rho_h) \in \boldsymbol{W}_h \times \boldsymbol{W}_h \times \boldsymbol{W}_h \times W_h$. These fields are known within the HDG as local fields, since they are defined within the elements T of the discretization. In addition, we introduce the additional variables $(\widehat{\mathbf{E}}_h, \widehat{\rho}_h) \in \boldsymbol{M}_h(\mathbf{0}|_{\Gamma_x}) \times M_h(0|_{\Gamma_x})$ that approximate the tangential component $(\mathbf{n} \times \mathbf{E} \times \mathbf{n})$ of $\mathbf{E}$ and the trace of ρ respectively. These fields, defined on the faces F of the discretization, are referred to as global variables. The weak formulation of the HDG method for the metal subdomain Ω_{m} reads

$$\begin{aligned} -i\omega(\mathbf{H}_h, \boldsymbol{\kappa})_{\mathcal{T}_{\mathrm{m}}} + (\mathbf{E}_h, \nabla \times \boldsymbol{\kappa})_{\mathcal{T}_{\mathrm{m}}} + \langle \widehat{\mathbf{E}}_h, \boldsymbol{\kappa} \times \mathbf{n} \rangle_{\partial\mathcal{T}_{\mathrm{m}} \setminus \Gamma_x} &= 0, \\ -\beta^2(\rho_h, \nabla \cdot \boldsymbol{\eta})_{\mathcal{T}_{\mathrm{m}}} + \beta^2 \langle \widehat{\rho}_h, \boldsymbol{\eta} \cdot \mathbf{n} \rangle_{\partial\mathcal{T}_{\mathrm{m}} \setminus \Gamma_x} + (\gamma - i\omega)(\mathbf{J}_h, \boldsymbol{\eta})_{\mathcal{T}_{\mathrm{m}}} - \omega_{\mathrm{p}}^2 (\mathbf{E}_h, \boldsymbol{\eta})_{\mathcal{T}_{\mathrm{m}}} &= 0, \\ (\mathbf{H}_h, \nabla \times \boldsymbol{\xi})_{\mathcal{T}_{\mathrm{m}}} + \langle \widehat{\mathbf{H}}_h, \boldsymbol{\xi} \times \mathbf{n} \rangle_{\partial\mathcal{T}_{\mathrm{m}}} + i\omega(\varepsilon_\infty \mathbf{E}_h, \boldsymbol{\xi})_{\mathcal{T}_{\mathrm{m}}} - (\mathbf{J}_h, \boldsymbol{\xi})_{\mathcal{T}_{\mathrm{m}}} &= 0, \\ i\omega(\rho_h, \zeta)_{\mathcal{T}_{\mathrm{m}}} - \langle \widehat{\mathbf{J}}_h \cdot \mathbf{n}, \zeta \rangle_{\partial\mathcal{T}_{\mathrm{m}}} + (\mathbf{J}_h, \nabla\zeta)_{\mathcal{T}_{\mathrm{m}}} &= 0, \\ \langle \widehat{\mathbf{J}}_h \cdot \mathbf{n}, \theta \rangle_{\partial\mathcal{T}_{\mathrm{m}} \setminus \Gamma_x} &= 0. \end{aligned} \tag{10}$$

The formulation for the dielectric subdomain Ω_{d} is given by

$$\begin{aligned} -i\omega(\mathbf{H}_h, \boldsymbol{\kappa})_{\mathcal{T}_{\mathrm{d}}} + (\mathbf{E}_h, \nabla\times\boldsymbol{\kappa})_{\mathcal{T}_{\mathrm{d}}} + \langle \widehat{\mathbf{E}}_h, \boldsymbol{\kappa}\times\mathbf{n}\rangle_{\partial\mathcal{T}_{\mathrm{d}}\setminus\Gamma_x} &= 0, \\ (\mathbf{H}_h, \nabla\times\boldsymbol{\xi})_{\mathcal{T}_{\mathrm{d}}} + \langle \widehat{\mathbf{H}}_h, \boldsymbol{\xi}\times\mathbf{n}\rangle_{\partial\mathcal{T}_{\mathrm{d}}} + i\omega(\varepsilon_{\mathrm{d}}\mathbf{E}_h, \boldsymbol{\xi})_{\mathcal{T}_{\mathrm{d}}} &= 0, \\ -\langle \mathbf{n}\times(\widehat{\mathbf{H}}_h - \mathbf{H}_0) + \sqrt{\varepsilon_{\mathrm{d}}}\times(\widehat{\mathbf{E}}_h - \mathbf{E}_0), \boldsymbol{\mu}\rangle_{\Gamma_{\mathrm{rad}}} &= 0. \end{aligned} \tag{11}$$

Additionally, we enforce continuity of the tangential component of the magnetic field $[\![\mathbf{n}\times\widehat{\mathbf{H}}]\!] = 0$ across all faces, as well as the PEC and PMC conditions on the lateral boundaries

$$-\langle \mathbf{n}\times\widehat{\mathbf{H}}_h, \boldsymbol{\mu}\rangle_{\partial\mathcal{T}\setminus\Gamma_x\cup\Gamma_{\mathrm{rad}}} = 0. \tag{12}$$

The above weak formulations hold for all $(\boldsymbol{\kappa}, \boldsymbol{\eta}, \boldsymbol{\xi}, \zeta, \boldsymbol{\mu}, \theta) \in \boldsymbol{W}_h\times\boldsymbol{W}_h\times\boldsymbol{W}_h\times W_h\times\boldsymbol{M}_h\times M_h$. The numerical fluxes are defined as

$$\begin{aligned} \widehat{\mathbf{H}}_h &= \mathbf{H}_h + \tau_t(\mathbf{E}_h - \widehat{\mathbf{E}}_h)\times\mathbf{n}, \\ \widehat{\mathbf{J}}_h\cdot\mathbf{n} &= \mathbf{J}_h\cdot\mathbf{n} - \tau_n i\omega(\rho_h - \widehat{\rho}_h). \end{aligned} \tag{13}$$

with $\tau_t = \sqrt{\varepsilon}$ and $\tau_n = \omega_{\mathrm{p}}/\beta$ are chosen through a dimensional analysis, and are defined globally to ensure the stability of the HDG discretization even in the presence of highly localized solutions. Plugging the numerical fluxes (13) into (10)-(11)-(12) and integrating by parts leads to the final form of the HDG discretization for a metallic nanostructure described with the hydrodynamic model, that is

$$\begin{aligned} -i\omega(\mathbf{H}_h, \boldsymbol{\kappa})_{\mathcal{T}_m} + (\mathbf{E}_h, \nabla\times\boldsymbol{\kappa})_{\mathcal{T}_m} + \langle \widehat{\mathbf{E}}_h, \boldsymbol{\kappa}\times\mathbf{n}\rangle_{\partial\mathcal{T}_m\setminus\Gamma_x} &= 0, \\ -\beta^2(\rho_h, \nabla\cdot\boldsymbol{\eta})_{\mathcal{T}_m} + \beta^2\langle \widehat{\rho}_h, \boldsymbol{\eta}\cdot\mathbf{n}\rangle_{\partial\mathcal{T}_m\setminus\Gamma_x} + (\gamma - i\omega)(\mathbf{J}_h, \boldsymbol{\eta})_{\mathcal{T}_m} - \omega_{\mathrm{p}}^2(\mathbf{E}_h, \boldsymbol{\eta})_{\mathcal{T}_m} &= 0, \\ (\nabla\times\mathbf{H}_h, \boldsymbol{\xi})_{\mathcal{T}_m} + \langle \tau_t[\mathbf{E}_h - \widehat{\mathbf{E}}_h], \mathbf{n}\times\boldsymbol{\xi}\times\mathbf{n}\rangle_{\partial\mathcal{T}_m} + i\omega(\varepsilon_\infty\mathbf{E}_h, \boldsymbol{\xi})_{\mathcal{T}_m} - (\mathbf{J}_h, \boldsymbol{\xi})_{\mathcal{T}_m} &= 0, \\ -(\nabla\cdot\mathbf{J}_h, \zeta)_{\mathcal{T}_m} + i\omega(\rho_h, \zeta)_{\mathcal{T}_m} + i\omega\tau_n\langle \rho_h, \zeta\rangle_{\partial\mathcal{T}_m} - i\omega\tau_n\langle \widehat{\rho}_h, \zeta\rangle_{\partial\mathcal{T}_m} &= 0, \\ -\langle \mathbf{n}\times\mathbf{H}_h, \boldsymbol{\mu}\rangle_{\partial\mathcal{T}_m\setminus\Gamma_x} - \langle \tau_t\mathbf{E}_h, \boldsymbol{\mu}\rangle_{\partial\mathcal{T}_m\setminus\Gamma_x} + \langle \tau_t\widehat{\mathbf{E}}_h, \boldsymbol{\mu}\rangle_{\partial\mathcal{T}_m\setminus\Gamma_x} &= 0, \\ \langle \mathbf{J}_h\cdot\mathbf{n}, \theta\rangle_{\partial\mathcal{T}_m\setminus\Gamma_x} - i\omega\tau_n\langle \rho_h, \theta\rangle_{\partial\mathcal{T}_m\setminus\Gamma_x} + i\omega\tau_n\langle \widehat{\rho}_h, \theta\rangle_{\partial\mathcal{T}_m\setminus\Gamma_x} &= 0, \end{aligned} \tag{14}$$

for the metal and

$$\begin{aligned} -i\omega(\mathbf{H}_h, \boldsymbol{\kappa})_{\mathcal{T}_d} + (\mathbf{E}_h, \nabla\times\boldsymbol{\kappa})_{\mathcal{T}_d} + \langle \widehat{\mathbf{E}}_h, \boldsymbol{\kappa}\times\mathbf{n}\rangle_{\partial\mathcal{T}_d\setminus\Gamma_x} &= 0, \\ (\nabla\times\mathbf{H}_h, \boldsymbol{\xi})_{\mathcal{T}_d} + \langle \tau_t[\mathbf{E}_h - \widehat{\mathbf{E}}_h], \mathbf{n}\times\boldsymbol{\xi}\times\mathbf{n}\rangle_{\partial\mathcal{T}_d} + i\omega(\varepsilon_{\mathrm{d}}\mathbf{E}_h, \boldsymbol{\xi})_{\mathcal{T}_d} &= 0, \\ -\langle \mathbf{n}\times\mathbf{H}_h, \boldsymbol{\mu}\rangle_{\partial\mathcal{T}_d\setminus\Gamma_x} - \langle \tau_t\mathbf{E}_h, \boldsymbol{\mu}\rangle_{\partial\mathcal{T}_d\setminus\Gamma_x} + \langle \tau_t\widehat{\mathbf{E}}_h, \boldsymbol{\mu}\rangle_{\partial\mathcal{T}_d\setminus\Gamma_x} - \sqrt{\varepsilon_{\mathrm{d}}}\langle \widehat{\mathbf{E}}_h, \boldsymbol{\mu}\rangle_{\Gamma_{\mathrm{rad}}} &= \langle \mathbf{f}_0, \boldsymbol{\mu}\rangle_{\Gamma_{\mathrm{rad}}}, \end{aligned}$$

$$\tag{15}$$

for the dielectric.

We then employ the corresponding basis functions on the elements and faces of the discretization $\mathcal{T}$ and obtain linear systems at the element level. For any metallic element $T \in \mathcal{T}_m$, the system reads

$$\left[\begin{array}{cccc|cc} -i\omega\mathbb{A} & 0 & \mathbb{B} & 0 & \mathbb{C} & 0 \\ 0 & (\gamma - i\omega)\mathbb{A} & -\omega_{\mathrm{p}}^2\mathbb{A} & -\beta^2\mathbb{P} & 0 & \beta^2\mathbb{O} \\ \mathbb{B}^\intercal & -\mathbb{A} & \mathbb{D} + i\omega\mathbb{A}_\varepsilon & 0 & -\mathbb{E} & 0 \\ 0 & -\mathbb{P}^\intercal & 0 & i\omega\mathbb{H} & 0 & -i\omega\mathbb{N} \\ \hline \mathbb{C}^\intercal & 0 & -\mathbb{E}^\intercal & 0 & \mathbb{M} & 0 \\ 0 & \mathbb{O}^\intercal & 0 & -i\omega\mathbb{N}^\intercal & 0 & i\omega\mathbb{T} \end{array}\right] \left[\begin{array}{c} \underline{\mathbf{H}} \\ \underline{\mathbf{J}} \\ \underline{\mathbf{E}} \\ \underline{\rho} \\ \hline \underline{\widehat{\mathbf{E}}} \\ \underline{\widehat{\rho}} \end{array}\right] = \left[\begin{array}{c} 0 \\ 0 \\ 0 \\ 0 \\ \hline 0 \\ 0 \end{array}\right], \tag{16}$$

whereas the system for any dielectric element $T \in \mathcal{T}_d$ reads

$$\left[\begin{array}{cc|c} -i\omega\mathbb{A} & \mathbb{B} & \mathbb{C} \\ \mathbb{B}^\intercal & \mathbb{D} + i\omega\mathbb{A}_\varepsilon & -\mathbb{E} \\ \hline \mathbb{C}^\intercal & -\mathbb{E}^\intercal & \mathbb{M} \end{array}\right] \left[\begin{array}{c} \underline{\mathbf{H}} \\ \underline{\mathbf{E}} \\ \hline \underline{\widehat{\mathbf{E}}} \end{array}\right] = \left[\begin{array}{c} 0 \\ 0 \\ \hline \mathbf{F}_0 \end{array}\right], \tag{17}$$

where $\mathbf{U}_T = [\underline{\mathbf{H}}, \underline{\mathbf{J}}, \underline{\mathbf{E}}, \underline{\rho}]$ and $\widehat{\mathbf{U}}_T = [\underline{\widehat{\mathbf{E}}}, \underline{\widehat{\rho}}]$ are the vectors containing the values of the corresponding fields at the degrees of freedom on the elements (local) and faces (global) respectively.

Invoking discontinuity of the approximation spaces enables the local elimination of $\mathbf{U}_T$, or local unknowns, thus expressing them as a function of only the degrees of freedom of the approximate traces $\widehat{\mathbf{U}}_T$, or global unknowns, for all the elements $T \in \mathcal{T}$. This static condensation strategy is a key component towards an efficient implementation of the HDG method. For an arbitrary element $T \in \mathcal{T}$ whose linear system is described by either (14) or (15), static condensation is algebraically computed as

$$\left[\begin{array}{c|c} \mathbb{U}_1 & \mathbb{U}_2 \\ \hline \mathbb{U}_3 & \mathbb{U}_4 \end{array}\right] \left[\begin{array}{c} \mathbf{U}_T \\ \hline \widehat{\mathbf{U}}_T \end{array}\right] = \left[\begin{array}{c} 0 \\ \hline \mathbf{u} \end{array}\right] \Rightarrow \begin{cases} \mathbf{U}_T = -\mathbb{U}_1^{-1}\mathbb{U}_2\widehat{\mathbf{U}}_T & \text{(18a)} \\ \left[\mathbb{U}_4 - \mathbb{U}_3\mathbb{U}_1^{-1}\mathbb{U}_2\right]\widehat{\mathbf{U}}_T = \mathbf{u} & \text{(18b)} \end{cases}$$

The first equation states the relation between local and global unknowns at the element level, whereas the second one represents the linear system involving only global unknowns. Since this procedure is performed element-wise the inversion of $\mathbb{U}_1$ is still a computationally efficient task, and it is trivially parallelizable across mesh elements. After performing static

condensation for all discretization elements, we need an additional compatibility condition to stitch the metallic and dielectric subdomains. For any two elements at the interface $T^+ \in \mathcal{T}_m$, $T^- \in \mathcal{T}_d$ that satisfy $T^+ \cap T^- \in \partial\Omega_\mathrm{m}$, each global degree of freedom on the face $F \in \partial T^- \cap \partial\Omega_\mathrm{m}$ needs three components $\{\widehat{\mathbf{E}}_h, \widehat{\rho}_h\}$, whereas on the remaining faces of F only two components $\{\widehat{\mathbf{E}}_h\}$ are required. Thus, after accounting for the different number of global components on the faces adjacent to the interfaces, we may assemble the global system of equations that comprises $\widehat{\mathbf{U}}$ degrees of freedom only, that is

$$\boldsymbol{\Upsilon}\widehat{\mathbf{U}} = \boldsymbol{\upsilon} \; . \tag{19}$$

The system above is typically smaller than standard frequency-domain DG methods, since instead of solving for 10 volume variables (resp. 6) in the metal (resp. dielectric), solves only for 3 surface variables (resp. 2), resulting in considerable savings. Finally, after computing $\widehat{\mathbf{U}}$ through direct solution we recover the values of the local variables element-wise for both metal and dielectric domains. This is efficiently achieved by reusing the Schur factors (18a) precomputed during static condensation.

To complete the images in the main text, we show the absolute values of the $\mathbf{E}_h, \mathbf{H}_h, \mathbf{J}_h$ fields for the 2 nm gap structure in Fig. 1 ($z = 0$ slice) and Fig. 2 ($x = y$ slice), corresponding to the $\lambda = 5.05$ μm (resonant frequency) and $b = 1.5$. The pattern of solutions suggest that an aggressive meshing style near the metal-insulator interface (see Fig. 2a), is required to properly capture the electromagnetic response of this device.

Supplementary Note 2: Gold thickness analysis

In this section, we analyze the impact of the gold thickness. This study was carried out with the purpose of ensuring the discrepancy between simulations in experiments observed for 1 and 2 nm gaps was not due to an erroneous gold thickness.

To that end, and starting from the nominal gold thickness 150 nm, nonlocal simulations were performed for 137.5, 125, 112.5 down to 100 nm thickness, as it was deemed unreasonable that the fabricated devices were thinner. The nonlocal b parameter was chosen 1.5, which lead to the best match between simulations and experiments, see Fig 4c-4d in the main text. Simulations enable us to infer a model that predicts the resonance location given the gap size and the gold thickness. Indeed, both the resonant wavelength and peak transmittance can be accurately regressed from the thickness using a second order polynomial, with an R^2 coefficient > 0.9999 for all gaps and quantities, see Fig. 3.

Additionally, transmittance values for 1, 5 and 10 nm gap were measured after 10 and 20 minutes of over-milling the structures. The results are shown in Fig. 4. For the 10 nm gap there is a good agreement in resonant wavelength, although the simulations predict a

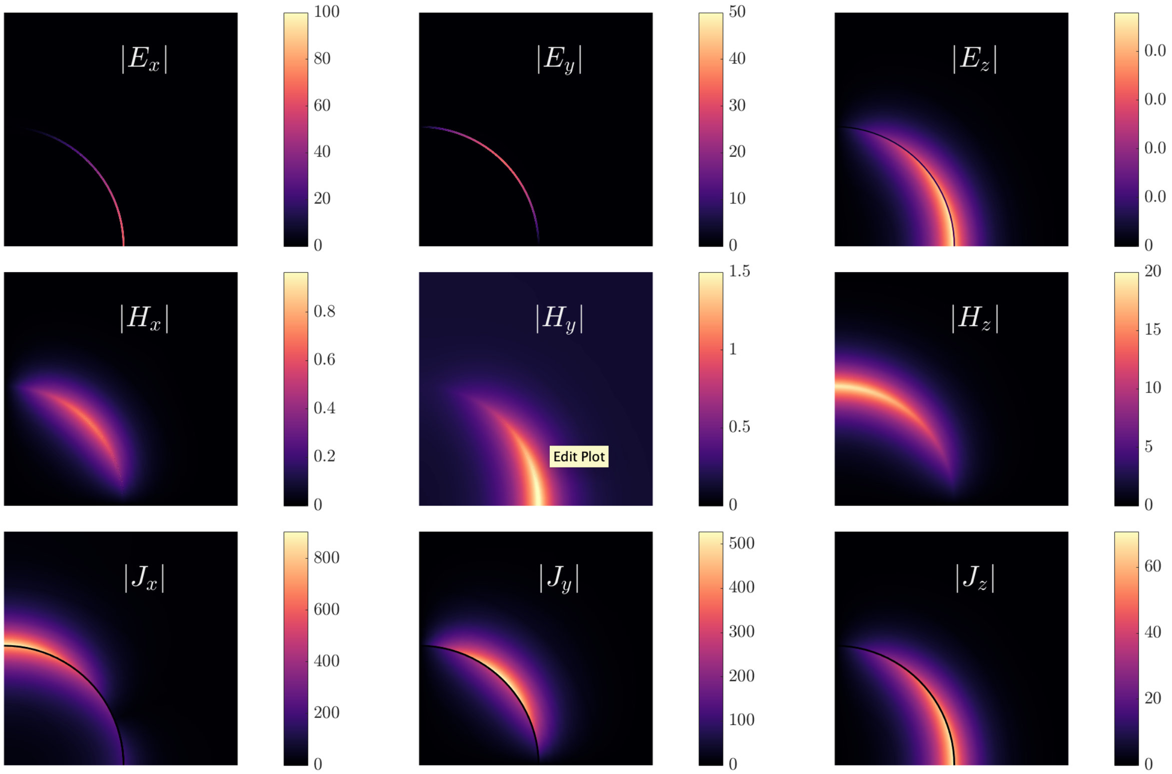

Supplementary Figure 1: Electric, magnetic and induced current fields at $z = 0$ for a 2 nm gap at $\lambda = 5.05\ \mu m$ and $b = 1.5$.

higher transmittance than experiments as we already saw in Fig. 4d, and this pattern is consistent for thinner films. For the 5 nm gap the experimental matching is significant both in resonant wavelength and transmittance. Tracking the resonance from the simulation data we can infer that the actual thickness after 10 min of over-milling is $\sim (121, 129)$ nm and after 20 minutes is $\sim (96, 107)$ nm, for the (10, 5) nm gap.

However, for the 1 nm gap not even a simulated gold thickness of 100 nm is able to fully bridge the shift observed between numerical calculations and experiments without over-milling. Hence, it is clear that the nonlocal model for 1 nm gap, which deliberately ignores quantum phenomena that become relevant at these scales, is insufficient to match the experimental data. Considering thinner films does not lead to better results, thus more accurate models that account for quantum effects are required.

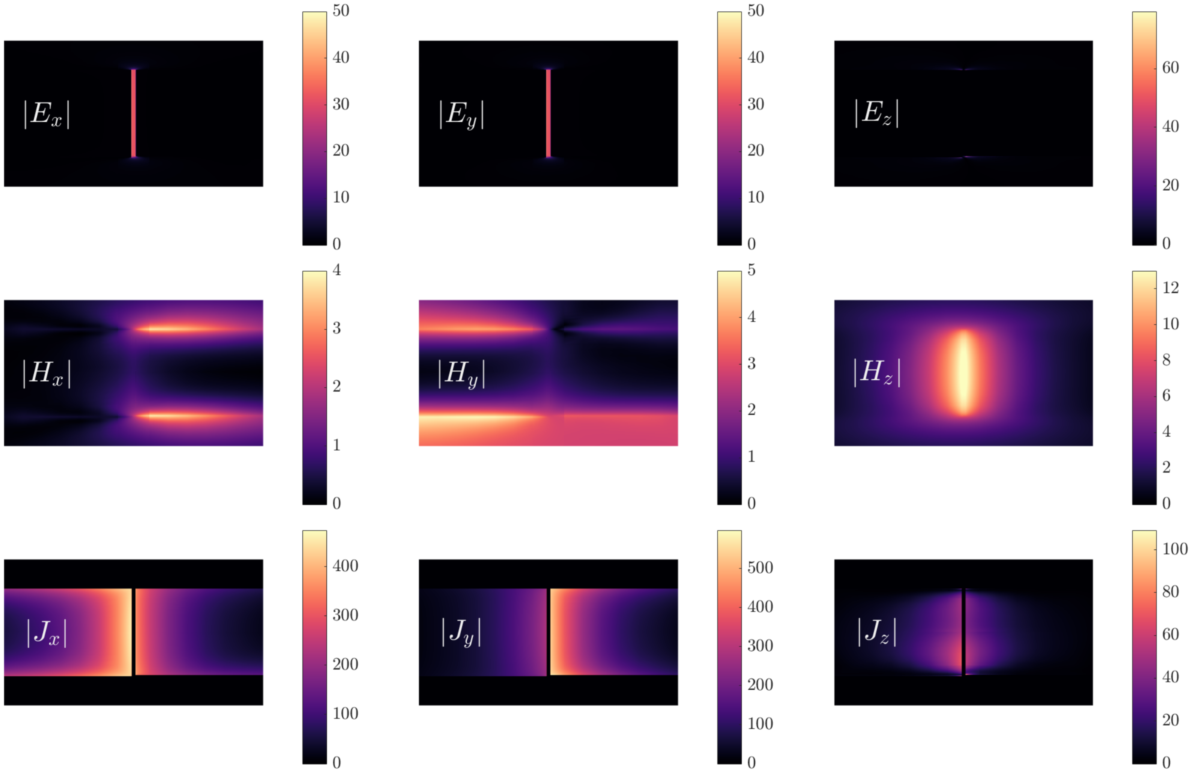

Supplementary Figure 2: Electric, magnetic and induced current fields at $x = y$ for a 2 nm gap at $\lambda = 5.05\ \mu m$ and $b = 1.5$.

Supplementary Note 3: Alumina thickness measurements

In our nanocoax device, the critical dimension that impacts nonlocality is the gap width, which in our process is defined by the thickness of ALD-grown Al_2O_3 films. Whenever Al_2O_3 layer was coated on the Au disk array patterns, a bare Si wafer was loaded together to monitor the film thickness. Using ellipsometry we measured 5 points (top, center, bottom, left, and right) on a bare Si wafer, and these measurements are provided in Table 1 for each nominal Al_2O_3 thickness. For convenience, the nominal thicknesses are used for figures, whereas the measured values are used for the simulations.

The inner diameter of a coaxial aperture D_{in} also largely determines the cutoff resonance of the structure. SEM images were taken from all samples, and the inner diameter of coax was measured using Image J analyzer. For each gap width, a total of 20 single coaxial apertures were measured, and the statistics are shown in Table 2.

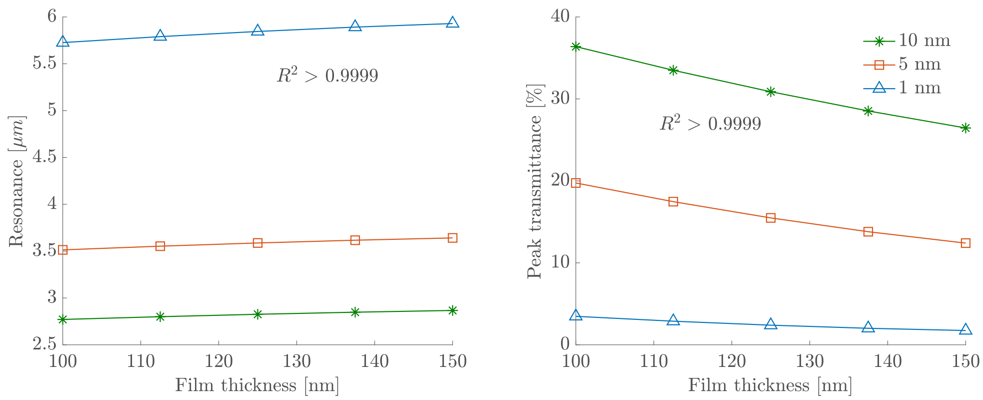

Supplementary Figure 3: Second-order relationships between thickness and resonance wavelength (left) and peak transmittance (right) computed with simulations results, for thicknesses between 100 and 150 nm.

	Nominal width (Å)						
	100	70	50	40	30	20	10
Measurement	Measured width (Å)						
1	95	71	48	39	20	20	11
2	93	72	49	40	30	19	10
3	95	70	48	40	31	19	11
4	96	70	49	41	32	18	10
5	95	71	49	39	31	19	12
Average (Å)	94.8	70.8	48.6	39.8	30.6	19	10.8
Range (Å)	3	2	1	2	3	2	2

Supplementary Table 1: Comparison of nominal and measured thin-film Al_2O_3 thickness.

	Nominal width (nm)						
	10	7	5	4	3	2	1
Statistics	Measured inner diameter (nm)						
Mean	252.70	251.35	255.00	254.05	252.05	253.65	251.95
Std	4.33	4.89	5.31	5.42	5.89	5.97	7.15
Min	241	240	244	240	238	239	237
Max	259	260	264	262	262	266	266

Supplementary Table 2: Statistics of measured inner diameter D_{in} of a coaxial aperture.

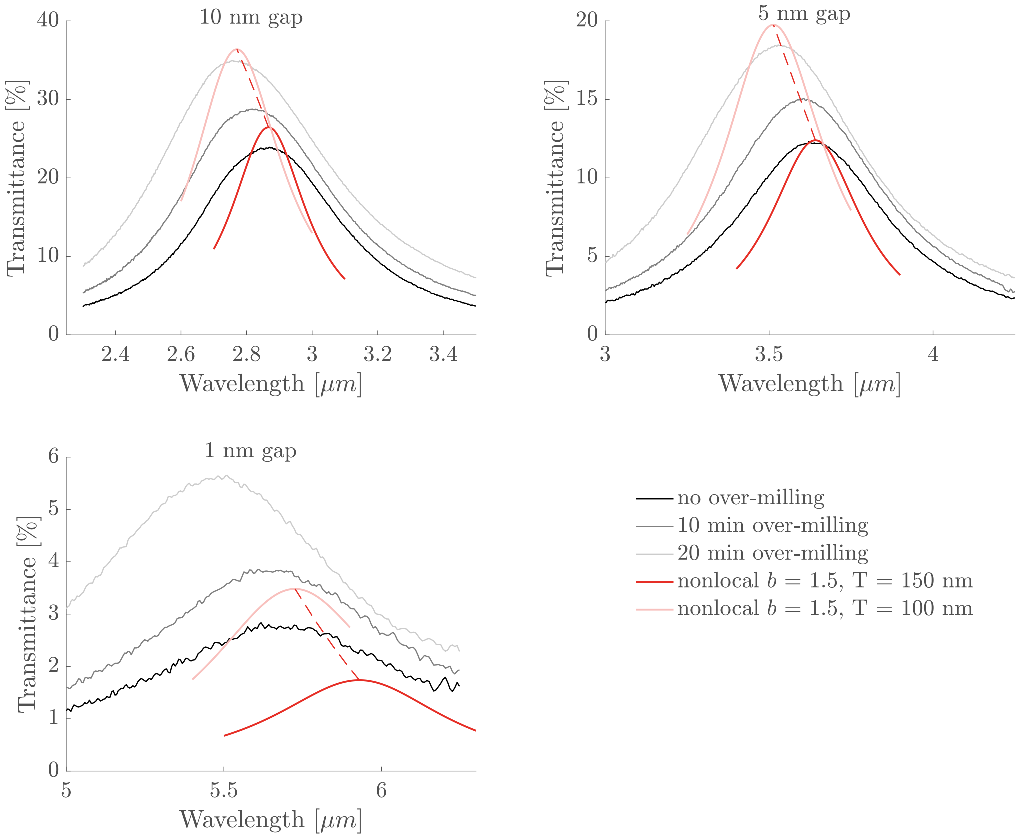

Supplementary Figure 4: Resonances of 1, 5 and 10 nm gap for experiments (with over-milling) and simulations (with thickness dependence).
